# Factors associated with quality of life among joint and nuclear families: a population-based study

**DOI:** 10.1186/s12889-021-10265-2

**Published:** 2021-01-28

**Authors:** Fahad Saqib Lodhi, Unaib Rabbani, Adeel Ahmed Khan, Owais Raza, Kourosh Holakouie-Naieni, Mehdi Yaseri, Umer Farooq, Ali Montazeri

**Affiliations:** 1 Department of Community Medicine, Women Medical and Dental College, Abbottabad, Pakistan; 2 Department of Epidemiology and Biostatistics, School of Public Health, Tehran University of Medical Sciences, Tehran, Iran; 3 Family Medicine Academy, Qassim, Kingdom of Saudi Arabia; 4 Saudi Board Program of Preventive Medicine, Ministry of Health, Mecca, Kingdom of Saudi Arabia; 5 School of Public Health, Dow University of Health Sciences, Karachi, Pakistan; 6 Community Medicine Department, Ayub Medical College, Abbottabad, Pakistan; 7 Population Health Research Group, Health Metrics Research Center, Iranian Institute for Health Sciences Research, ACECR, Tehran, Iran; 8 Faculty of Humanity Sciences, University of Science and Culture, Tehran, Iran

**Keywords:** Quality of life (QOL), Family system, WHOQOL-BREF, General population, Pakistan

## Abstract

**Background:**

Advantages and disadvantages associated with joint and nuclear family systems can affect quality of life (QOL). However, there is scarcity of literature about QOL among joint and nuclear family systems. This study aimed to assess the factors associated with QOL in joint and nuclear family systems.

**Methods:**

We conducted a population based cross sectional study in all 52 Union Councils (UCs) of District Abbottabad, Khyber Pakhtunkhwa province, Pakistan from March 2015 to August 2015. Multistage cluster sampling technique was used to select participants from both nuclear and joint family houses. The validated Urdu version of World Health Organization Quality of Life Questionnaire-Brief Version (WHOQOL-BREF) was used to assess quality of life among participants. Univariate and multivariate analyses were performed to explore the associations of different socio demographic variables with QOL among both family systems. Also a multilevel linear regression using backward analysis to obtain final model for each domain was performed to find out the variables that are associated with QOL score in each of family systems.

**Results:**

A total of 2063 participants were included in this study (51.0% joint family, 49.0% nuclear family) with the response rate of 97.4%. In multiple linear regression analysis of each domain for joint and nuclear family systems, rural residence compared to urban (*p* < 0.001), being female (*p* < 0.001), older age (*p* < 0.001), having comorbidity (*p* < 0.001) and lower socioeconomic status (*p* < 0.001) were found to be a strong predictor of poorer QOL. Furthermore, social capital (*p* < 0.001) had a positive effect on joint and nuclear family QOL scores.

**Conclusion:**

This study was the first of its kind which determined the factors of QOL in joint and nuclear families using the validated Urdu version of WHOQOL-BREF in Pakistan. Male gender, urban residence, younger age, higher socioeconomic status and social capital were positive predictors of QOL score while older age and presence of illness were associated with lower QOL scores among both family systems.

## Introduction

In general family is one of the fundamental units of societies and takes care of the diverse needs of people [1]. It is also one of the basic sources of providing care to all of its members. Because of this fact elderly persons of the house occupy respectful position in Asian culture. Family system encourages the life of individuals in all aspects which enables them to live happy and productive life [2]. Culture has been shown to regulate the family network by building family type, family size and form [3, 4] and the family functioning by defining barriers, cooperation rules, connection patterns, adequate practices, regulation and ranking in the family [4–7].

Family is a social group of one or more parents and their children. Family systems refer to members and their interrelationship (structure) with each other. There are different classifications of family systems [8, 9]. Most commonly used classification has two types i.e. joint and nuclear family systems [10]. A nuclear family system is defined as ‘a two generation family consisting of a father and mother and children or a single, possibly widow, parent and his/her children’ [11]. Similarly, joint or extending family is defined as ‘three or more generations lived together with both vertical and lateral extension having a single line of authority, either patrilineal or matrilineal’ [11]. A number of advantages and disadvantages associated with each type of family has been reported such as social support, protection during crises, physical space, autonomy a freedom of decision making [12].

Extent of these systems varies from countries to countries and within countries as well. Traditionally Pakistan had joint family system and bonding within a family. Like other Asian countries, over the time, balance is shifting towards nuclear family system in Pakistan [13]. Multiple factors are responsible for this shifting trend from joint to nuclear system. These include; financial pressures, decreasing living space, movement for job and rapid urbanization [13]. It also seems to be an outcome of increasing prosperity. This trend is faster in urban areas than rural areas. The superiority of one of these systems is a matter of debate these days. The researchers are on a quest for evidence based information regarding the current debate about the quality of life of an individual, based on a family system [14].

In Pakistan, a large number of the aged people depend on their family especially on their children or grandchildren for physical, communal and financial support [2] which is more convenient in joint families. It was recommended by Mason (1992) that urbanization is expected to negatively affect the family’s capacity and willingness for care of the elderly and it will also decrease the chances of living grown up children with their parents [15]. Studies from Asian countries have shown that most of the help for the elderly people comes from their home by their children/grandchildren [16, 17].

Limited studies have been conducted on different study populations that have assessed the predictors of quality of life. A study conducted among elderly population in India reported that occupation, higher income, 60–69 years age group, staying with partner and absence of co-morbidity were found to be the determinants of better QOL [18]. Studies from Kuwait and Lebanon also reported that female gender, older age, social disadvantage, and presence of anxiety/ depression were associated with poor QOL [19, 20]. Although all of the works done before were on health-related QOL all around the world, there are no such study exploring the predictors of quality of life of people who live in nuclear or joint family system. Our study presented the predictors of quality of life scores in joint and nuclear family systems in Pakistani general population.

## Methods

### Study design and setting

We conducted a population based cross sectional study in all 52 Union Councils of District Abbottabad, Khyber Pakhtunkhwa province, Pakistan from March 2015 to August 2015. We recruited 2063 participants for our study. Abbottabad is the main district of Khyber Pakhtunkhwa province of Pakistan having more than 1.2 million population living in 52 union councils. The primary language spoken here is Hindko (used by 94% of the rural population and 75% of urban residents) followed by Urdu which is also spoken and understood in rural & urban areas [21].

### Sample size

We used the Statulator, an online statistical calculator for sample size determination [22]. Assuming a standard deviation of 12 units (derived from pilot study) and a design effect (DEFF) of 2, the study would require a sample size of 969 for each group (i.e. a total sample size of 1938, assuming equal group sizes), to achieve a power of 90% at 5% significance level (two sided) for detecting a true difference of 2.5 points in quality of life score between joint and nuclear family systems.

### Sampling procedure

Participants were selected from all union councils (UCs) of District Abbottabad. Multistage cluster sampling technique was employed in this study. Each union council was further divided into several blocks called Mohallah. We did proportionate sampling according to the 1998 population census [23] of UCs for the selection of Mohallah & on the next stage households. In the first stage we randomly selected these blocks (Mohallah) in each of the UC from a list by using simple random sampling technique. In the next stage we selected households in that selected block by using a random sampling technique again. The total number of houses selected in each block was also proportional to the population size of respective block. For the selection of family type, from the list of household of each block, we made a list of joint & nuclear family system households and enrolled equal number of houses from both family types. A simple random sampling technique was used for the selection of person (≥18 years) from each house. Simple random sampling was done by applying the lottery method for selecting the ≥18 year’s participant for the study. The inclusion criteria used for selection of individual were age greater than 18 years and permanent resident of union council for at least 5 years. Guests and temporary residents were excluded from the study.

### Measures

1. We used the World Health Organization Quality of Life Questionnaire-Brief version (WHOQOL-BREF) for measuring quality of life. It is in public domain and contains 26-items that covers four domains of QOL (psychological 6 items, physical 7 items, social relationships 3 items and environmental 8 items). Each question scored on a scale from one to five, with high score indicating good QOL with the exception of three questions, which include pain and discomfort, need for medical treatment and negative feelings [24]. The seven items included in the physical health domain were mobility, daily life activities, pain, sleep, functional capacity and energy. The psychological domain measured negative thinking, self-image, positive approach, self-esteem, mindset, ability to learn, memory, consolidation, religion and the psychic conditions. Questions such as social support, sex-life and personal relationship come under the social relationship domain. The environmental health domain contains questions on financial assets, security, health and social services, living in natural environment, opportunities for advance learning experience, relaxation, and natural environment (air, noise, pollution and transportation) [25]. The total raw score for these four dimensions were transformed into 0 to 100 scale according to the standard procedure defined in WHO QOL user manual [24], and then analysis of this reconstruct score was done. Psychometric properties and validation of this WHOQOL-BREF questionnaire was done in the national language “Urdu”. The Cronbach’s alpha for each of four domains were 0.78, 0.71, 0.73 and 0.65, respectively [26]. To assess the feasibility and clarity of the items, a pilot study was conducted on 30 individuals conveniently selected from the study area.
2. We also developed a structured demographic questionnaire which included variables such as age, gender, marital status, type of family (joint and nuclear), residence type (urban and rural), house ownership (owner, not owner), respondent education (no education, madrassa, can read/write, primary- up to grade 5, secondary education-up to grade 12 and tertiary-up to grade 16 or above), working status (employed, unemployed and retired).
3. The socio-economic characteristics were assessed by taking household conditions, sources of drinking water, sanitation facilities, availability of electricity, housing facilitates, possession of durable goods, mean of transport, inventory of house hold and personal items such as chairs, clocks, buckets, radios, television sets, fans, stoves or cookers, cars, and telephones. This list was composed of 21 such items used in the Pakistan Demographic and Health Survey 2013 [27]. Wealth index was measured by an index constructed from principle component analysis (PCA) [28] of items indicating ownership of household durables and dwelling characteristics.
4. The World Bank’s Social Capital Integrated Questionnaire (SC-IQ) was also used to study social capital among families. It is an open-domain questionnaire and consists of 27 questions in six domains [29]. Of these five questions on overall trust, trust in local government, trust in central government, community cooperation and safety at home were selected and used in this study. These questions were translated by the research team into Urdu and then back translated into English by independent bilingual expert to assess the validity of the translation. The internal consistency of the items as assessed by the Cronbach’s alpha coefficient was found to be acceptable (alpha = 0.64). The concurrent validity as assessed by correlation between the trust subscale of the SC-IQ and the social relationship of the WHOQOL-BREF showed satisfactory result (r = 0.74).

### Data collection

One-day training session was conducted for administering the questionnaires prior to data collection for lady health workers of all UCs by principal investigator. In 1994, Pakistan’s Ministry of Health (MOH) implemented the Lady Health Worker Program (LHWP) as part of a national strategy to reduce poverty and improve health by bringing health services to the doorsteps of underserved communities. Lady health workers are out reach health workers who provide preventive and health promotion services specially for maternal and child health issues [30]. The questionnaire was administered through face to face interviews in the households by trained lady health workers of that union council. To ensure privacy and confidentiality, interviews were conducted in an independent room or area separate from other members of the family.

### Statistical analysis

The data was analyzed using the Stata version 13.0 (Stata Corp, College Station, TX, USA). First, we conducted descriptive analyses such as frequencies, proportions and means. Then, we carried out univariate linear regression analyses with domain scores as dependent and other variables as independent variables. Next, in the multivariate analysis, we included all independent variables and used stepwise backward approach to eliminate variables with a *p* value > 0.05. Finally, multi-level analysis was performed with two –level continuous random intercept model with individuals nested within clusters was applied to explore the variability explained by individuals and cluster level variables taking the correlated nature of data into account. *P*-value of < 0.05 was considered as significant.

## Results

### Demographic characteristic of study participants

A total of 2116 households were approached. Of these, 56 refused (non-response 2.64%) to participate in this study giving a total number of 2063 [1053 (51.04%) belonged to joint family system and 1010 (48.6%) to nuclear family]. Younger (18–30 years) and elderly (> 50 years) were more in joint families (39%) and (20%) respectively compared to (20%) and (10%) in nuclear families. Educational status of the participants was comparable in two groups as proportions of individuals with no education were 15.5% and 15.7 in joint and nuclear families respectively. Majority were living in their own houses 849 (80.5%) in joint and 750 (74.3%) in nuclear family. A higher proportion of the participants (80.5%) in joint family groups owned a house compared to (71%) in nuclear family group. Proportions of employed persons were (56.5%) in joint families and (56.1%) in nuclear families. Higher proportion of the participant were satisfied in living in joint family system (87.5%) compared to (81%) in nuclear family system (Table 1).

**Table 1:** Socio-demographic characteristic of the study participants (*n* = 2063)

	Joint family system (***n*** = 1053)	Nuclear family system (***n*** = 1010)	All (***n*** = 2063)
	No. (%)	No. (%)	No. (%)
**Age**			
18–30	412(39.2)	308(20.6)	720(34.9)
31–40	277(26.3)	359(35.4)	636(30.8)
41–50	149(14.2)	240(23.7)	389(18.8)
> 50	215(20.4)	103(10.2)	318(15.4)
**Gender**			
Males	553 (52.5)	505 (50)	1078 (52.2)
Females	500 (47.5)	505 (50)	1005(48.7)
**Marital status**			
Married	843 (80.0)	796 (78.8)	1639 (79.4)
Widowed/Widower	36 (3.4)	24 (2.37)	60 (2.9)
Divorced	4(0.3)	2 (0.1)	6 (2.0)
Separated	5(0.4)	4(0.3)	9 (0.4)
Never Married	165 (15.6)	184(18.2)	349(16.9)
**Education**			
No education	163(15.4)	159(15.7)	322(15.6)
Informal education	17(1.6)	30(2.9)	47(2.2)
Can read / write	111(10.5)	100(9.9)	211(10.2)
Primary (up to grade 1)	344(32.6)	293(29)	637(30.8)
Secondary (up to grade 2)	321(30.4)	337(33.3)	658(31.8)
Tertiary (up to grade 3)	97(9.2)	91(9)	188(9.1)
**Place of residence**			
Urban	297(28.3)	301(29.8)	598(29)
Rural	756 (71.7)	709(70.2)	1465(71)
**Ownership of residence**			
Owner	849(80.5)	750(74.3)	1599(77.5)
Not owner	204(19.5)	259(25.7)	463(22.4)
**Occupation**			
Not working	397 (37.7)	413(40.9)	810(39.3)
Working	595 (56.5)	567(56.1)	1162(56.3)
Retired	61 (5.8)	30(3)	91(4.4)
**Socioeconomic status**			
High	391(37.1)	296(29.3)	687(33.3)
Intermediate	375(35.6)	315(31.2)	690(33.4)
Low	287(27.3)	399(39.5)	686(33.3)
**Respondent disease**			
Physical disability	13(1.2)	15(1.5)	28(1.4)
Hypertension	79(7.5)	75 (7.4)	154(7.5)
Diabetes	34(3.2)	24(2.4)	58(2.8)
Other	492 (47)	488(48.3)	980(47.5)
None	435(41.3)	408(40.4)	843(41)
**Respondent satisfaction**			
Satisfied	921(87.5)	817(81)	1738(84.2)
Unsatisfied	132(12.5)	193(19)	325(15.8)
**Social Capital**			
Low	96(9.1)	118(11.7)	214(10.4)
Moderate	816(77.5)	755(74.7)	1571(76.1)
High	141(13.4)	137(13.6)	278(13.5)

### Joint family system WHO QOL-BREF scores

Table 2 shows mean score of WHOQOL-BREF scores of participants in joint families. Those living in the urban areas, had significantly higher scores in all four dimensions. Male had higher scores in physical and psychological domains compared to females. However, no differences were observed in relationship and environmental domains. Younger age group < 30 years had higher scores than elderly > 50 years of age. Divorced had highest scores in physical and psychological domains while married had highest scores in relationship domain. No significant differences were observed in environment domain between different categories of marital status. Lack of education, presence of any physical disability or disease, unemployment, lower socio-economic status and low social capital were associated with lower scores in all domains.

**Table 2:** QOL scores among different subgroups in joint family system, Abbottabad, Pakistan (*n* = 1053)*

	Physical	Psychological	Relationship	Environmental	General facet
	Mean ± SD	Mean ± SD	Mean ± SD	Mean ± SD	Mean ± SD
**Type of residence**					
Urban	68.6 ± 15.6	71.0 ± 14.4	74.1 ± 15.8	59.9 ± 14.2	69.4 ± 16.0
Rural	63.7 ± 14.9	65.9 ± 14.7	71.0 ± 16.6	53.7 ± 14.5	69.2 ± 16.9
*P value (t test)*	*< 0.001*	*< 0.001*	*< 0.001*	*< 0.001*	*0.891*
**Gender**					
Male	66.7 ± 15.6	69.0 ± 14.5	72.4 ± 16.8	56.5 ± 13.6	68.6 ± 17.3
Female	63.4 ± 14.6	66.6 ± 14.2	72.3 ± 17.0	56.5 ± 14.2	70.0 ± 15.8
*P value (t test)*	*< 0.001*	*< 0.006*	*0.896*	*0.988*	*0.161*
**Age**					
< 30	67.8 ± 14.0	68.7 ± 13.4	73.8 ± 15.4	56.9 ± 14.00	70.4 ± 15.4
31–40	65.3 ± 14.6	68.4 ± 15.1	72.1 ± 17.0	56.0 ± 14.3	69.7 ± 17.1
41–50	64.3 ± 16.0	68.2 ± 13.8	72.0 ± 18.0	57.0 ± 15.3	69.4 ± 17.7
> 50	60.4 ± 16.8	65.1 ± 15.3	69.7 ± 18.6	56.0 ± 14.4	66.4 ± 17.4
*P value (F statistic)*	*< 0.001*	*0.020*	*0.038*	*0.829*	*0.041*
**Residence ownership**					
Owner	65.0 ± 15.2	67.7 ± 14.21	72.6 ± 16.8	56.5 ± 14.3	68.3 ± 17.2
Not owner	66.1 ± 15.1	70.2 ± 16.4	70.1 ± 16.5	58.2 ± 15.0	69.6 ± 18.5
*P value (t test)*	*0.590*	*0.208*	*0.276*	*0.340*	*0.361*
**Marital status**					
Married	64.6 ± 15.0	67.8 ± 14.4	73.0 ± 16.9	56.5 ± 14.4	69.3 ± 16.7
Widow	56.2 ± 15.7	60.4 ± 15.2	65.5 ± 17.2	52.0 ± 13.9	61.4 ± 18.3
Divorced	76.8 ± 10.7	76.0 ± 10.4	60.4 ± 14.2	53.1 ± 6.8	65.6 ± 12.0
Separated	69.6 ± 20.7	63.5 ± 15.0	68.7 ± 17.2	52.3 ± 8.6	65.6 ± 21.3
Never married	69.4 ± 14.8	70.0 ± 13.6	71.0 ± 16.3	57.9 ± 14.1	71.3 ± 15.3
*P value (F statistic)*	*< 0.001*	*0.006*	*0.038*	*0.227*	*0.029*
**Education**					
No education	59.5 ± 15.8	62.9 ± 16.0	67.8 ± 19.4	54.5 ± 15.2	62.3 ± 19.0
Informal education	65.4 ± 19.5	71.1 ± 16.0	72.9 ± 14.7	58.8 ± 17.1	71.1 ± 13.5
Can read / write	63.0 ± 16.6	63.9 ± 14.9	68.6 ± 18.9	55.3 ± 13.8	66.2 ± 16.8
Primary (up to grade 5)	66.0 ± 14.5	68.1 ± 13.4	73.3 ± 16.2	57.2 ± 13.6	69.2 ± 16.2
Secondary (up to grade 12)	67.5 ± 14.8	71.1 ± 13.7	74.0 ± 15.3	57.2 ± 14.0	73.1 ± 15.0
Tertiary (up to grade 16 or above)	63.3 ± 14.0	68.8 ± 13.1	75.7 ± 16.0	56.4 ± 14.7	71.8 ± 16.1
*P value (F statistic)*	*< 0.001*	*< 0.001*	*< 0.001*	*0.317*	*< 0.001*
**Disease**					
Physical disability	58.8 ± 17.5	61.8 ± 18.1	69.2 ± 12.9	51.7 ± 13.5	63.5 ± 14.8
Hypertension	61.3 ± 18.0	65.2 ± 15.4	79.0 ± 18.0	57.0 ± 15.8	65.7 ± 17.8
Diabetes	57.0 ± 19.2	61.8 ± 18.8	69.4 ± 19.3	56.5 ± 15.6	59.2 ± 24.9
Other	64.0 ± 15.1	66.8 ± 14.0	72.0 ± 17.1	55.1 ± 14.2	69.2 ± 16.4
None	68.0 ± 13.8	70.1 ± 13.8	73.7 ± 16.1	58.2 ± 13.8	71.1 ± 15.5
*P value (F statistic)*	*< 0.001*	*< 0.001*	*0.095*	*0.017*	*< 0.001*
**Employment status**					
Not working	62.8 ± 16.3	66.3 ± 14.5	72.0 ± 16.9	56.4 ± 14.4	66.5 ± 17.4
Working	66.8 ± 14.0	69.0 ± 14.8	72.4 ± 17.0	56.5 ± 14.3	71.3 ± 15.8
Retired	63.0 ± 18.3	67.5 ± 14.4	74.3 ± 17.0	59.0 ± 14.9	68.1 ± 17.0
*P value (F statistic)*	*< 0.001*	*0.016*	*0.605*	*0.402*	*< 0.001*
**Socioeconomic status**					
High	67.8 ± 15.3	71.5 ± 13.2	76.5 ± 15.1	61.2 ± 13.3	74.3 ± 15.3
Intermediate	65.6 ± 15.1	68.4 ± 14.1	73.1 ± 15.6	56.9 ± 13.5	69.1 ± 15.4
Low	60.7 ± 15.3	62.2 ± 14.6	65.8 ± 18.7	50.0 ± 14.4	62.8 ± 17.6
*P value (F statistic)*	*< 0.001*	*< 0.001*	*< 0.001*	*< 0.001*	*< 0.001*
**Social capital**					
High SC	67.1 ± 14.9	71.3 ± 13.7	76.8 ± 16.1	59.8 ± 14.0	74.7 ± 14.5
Moderate SC	65.2 ± 15.1	67.8 ± 14.7	72.4 ± 16.5	56.4 ± 14.2	69.0 ± 16.3
Low SC	62.3 ± 16.4	64.4 ± 14.4	66.3 ± 18.2	50.9 ± 16.6	64.1 ± 19.9
*P value (F statistic)*	*0.059*	*< 0.001*	*< 0.001*	*0.005*	*< 0.001*

*Using the 0–100 scoring method

### Multivariate linear regression model for joint family system

Table 3 shows the results of the multivariate linear regression model for joint family system. In multivariate model, rural residence was negatively associated with physical, psychological and environmental domains QOL scores and there was 4.59 units [95% CI: − 7.77 to − 1.41], 3.54 units [95% CI: − 6.44 to − 0.63] and 5.32 units [95% CI: − 8.75 to - 1.89] reduction when changing from urban to rural (*P* = 0.001) respectively. There was no significant association with relationship domain. Female gender was also negatively associated with QOL scores in physical, psychological and social relationship domains − 3.94 units [95% CI: − 5.88 to − 0.01], − 3.10 units [95% CI: − 4.7 to − 1.47] and − 0.11 units [95% CI:-3.06 to- 0.84] respectively, and no significant association was observed in environmental health domain. Increasing age was negatively associated with QOL scores. One-decade increase in age lead to 0.22 units [95% CI: − 0.29 to − 0.19], 0.11 units [95% CI: − 0.2 to - 0.05] and 0.12 units [95% CI: − 0.19 to − 0.05] reduction in scores of physical, psychological and relationship domains respectively. Presence of disease was also significantly associated as scores declined with the presence of disease in physical and psychological domains. However, there was no significant association of disease status with relationship and environment domains. Socio-economic status also had a significant association as a change in SES from high to low resulted in a reduction of QOL scores in all the domains. Similarly increase in social capital was also positively associated with QOL scores in all four domains.

**Table 3:** Multivariate linear regression analysis for physical, psychological, social and environmental health domains of joint family in Abbottabad, Pakistan (*n* = 1053)*

	Physical Domain	Psychological Domain	Relationship Domain	Environmental Domain
	β (95% CI)	β (95% CI)	β (95% CI)	β (95% CI)
*Fixed effects*				
**Residence**				
Urban	Ref.	Ref.	Ref.	Ref.
Rural	−4.59(−7.77 to −1.41)	−3.54(−6.4 to −0.63)	–	−5.32(−8.75 to − 1.89)
**Gender**				
Male	Ref.	Ref.	Ref.	Ref.
Female	−3.94(−5.88 to −2.01)	−3.10(−4.7 to −1.47)	−1.11(− 3.06 to −0.84	–
**Age** (Decades)	−0.22(− 0.29 to − 0.19	−0.11(− 0.2 to − 0.05)	−0.12(− 0.19 to − 0.05)	–
**Disease**				
None	Ref.	Ref.	Ref.	Ref.
Physical disability	−10.3(− 17.8 to − 2.80)	− 8.79(− 15.8 to − 1.7)	–	–
Hypertension	−5.26(−9.3 to − 1.85	−5.26(− 8.87 to − 1.7)	–	–
Diabetes	−8.05(− 13.13 to − 2.9)	−6.17(− 10.9 to − 1.3)	–	–
Others	−4.26(− 6.59 to − 1.94)	− 3.36(− 5.5 to − 1.18)	–	–
**Socio-economic status**				
High	Ref.	Ref.	Ref.	Ref.
Intermediate	−0.33(−2.39 to 1.73)	− 2.20(− 4.1 to − 0.25)	−3.09(− 5.37 to − 0.80)	−3.76(− 5.61 to − 1.90)
Low	− 3.81(− 6.17 to − 1.45)	− 7.60(− 9.8 to − 5.40)	−9.86(− 12.4 to − 7.30)	−9.06(− 11.2 to − 6.94)
**Social capital**	0.12(0.06 to 0.17)	0.13(0.08 to 0.19)	0.16(0.10 to 0.22)	0.13(0.08 to 0.18)
*Random-effects*				
**Level 1: Union Council**				
Gender	0.02(0.00 to 4.54)	0.97(0.24 to 3.95)	0.43(0.21 to 4.53)	2.44(1.38 to 4.30)
Age	0.02(0.00 to 6.64)	0.05(0.00 to 0.51)	0.06(0.02 to 0.18)	0.05(0.01 to 0.24)
Social capital	0.04(0.01 to 0.21)	0.06(0.03 to 0.11)	0.02(0.00 to 1.22)	0.02(0.00 to 1.39)
Constant	3.0(1.02 to 8.81)	0.01(0.00 to 0.48)	0.01(0.00 to 0.20)	0.99(0.52 to 10.86)
**Level 2: Cluster number**				
Constant	3.30(2.04 to 5.36)	4.20(3.07 to 5.75)	5.50(4.15 to 7.31)	5.10(3.96 to 6.57)
Residual	13.07(12.4 to 13.71)	12.14(11.5 to 12.7)	14.6(13.97 to 15.34)	11.5(10.93 to 12.0)

*Explanations: *Β* Beta coefficient, *CI* Confidence interval, *Ref* Reference group; The linear regression multivariate model adjusted for residence ownership, marital status, education and employment status in Physical domain and Psychological domain. Type of residence, residence ownership, marital status, education, employment status and disease for Social domain; Residence ownership, marital status, age, sex, education, employment status, and disease in Environmental domain; The short dashes (−) mean that the variable was removed by the stepwise deletion process in regression analysis

### Nuclear family system WHOQOL-BREF scores

The mean score of each domain among different subgroups in joint family system is presented in Table 4. Pattern of differences between the subgroups in nuclear family system was similar to joint family system. The mean of all four domains was significantly higher among those living in urban areas. Male had higher scores than female. Younger age people < 30 years of age had significantly higher scores than elderly in physical domain only. There were no significant differences in other three domains with respect to age. House ownership did not affect the QOL scores in any of the domain. Significant differences in scores were observed across different marital status strata. Those with no education generally had lower scores than others. Presence of any disease or disability significantly reduced the QOL scores. Compared with working/employed subjects, unemployed subjects had lower QOL scores. Participants with higher socioeconomic status, and social capital levels had higher QOL scores in all domains.

**Table 4:** QOL scores among different subgroups in nuclear family system, Abbottabad, Pakistan (*n* = 1010)*

	Physical	Psychological	Relationship	Environmental	General facet
	Mean ± SD	Mean ± SD	Mean ± SD	Mean ± SD	Mean ± SD
**Type of residence**					
Urban	67.4 ± 15.8	70.5 ± 15.2	73.0 ± 16.0	58.5. ± 14.8	66.6 ± 18.5
Rural	63.7 ± 14.8	65.5 ± 15.2	70.8 ± 16.0	52.6 ± 15.0	67.1 ± 18.6
*P value (t test)*	*< 0.001*	*< 0.001*	*< 0.001*	*< 0.001*	*0.714*
**Gender**					
Male	66.0 ± 15.6	68.4 ± 14.4	72.5 ± 14.9	55.6 ± 14.7	66.0 ± 18.9
Female	63.6 ± 14.8	65.5 ± 16.1	70.4 ± 17.2	53.2 ± 15.5	68.0 ± 18.1
*P value (t test)*	*< 0.001*	*< 0.003*	*0.037*	*0.011*	*0.079*
**Age**					
< 30	67.8 ± 14.4	68.3 ± 15.0	69.6 ± 16.8	54.5 ± 15.4	68.8 ± 17.9
31–40	64.2 ± 14.0	66.0 ± 15.1	72.4 ± 15.7	53.4 ± 14.8	66.8 ± 18.2
41–50	64.0 ± 15.2	67.7 ± 15.3	72.5 ± 15.5	55.4 ± 14.1	65.7 ± 18.2
> 50	60.4 ± 17.3	64.6 ± 16.9	71.2 ± 15.9	54.5 ± 17.6	64.7 ± 21.6
*P value (F statistic)*	*< 0.001*	*0.079*	*0.87*	*0.476*	*0.128*
**Residence ownership**					
Owner	64.8 ± 15.0	66.9 ± 15.1	71.3 ± 15.9	54.1 ± 15.0	68.3 ± 17.2
Not owner	65.3 ± 17.0	67.8 ± 17.1	72.3 ± 17.5	54.4 ± 16.2	69.6 ± 18.5
*P value (t test)*	*0.700*	*0.560*	*0.509*	*0.818*	*0.361*
**Marital status**					
Married	63.9 ± 14.7	67.2 ± 15.0	72.6 ± 15.3	54.5 ± 14.9	67.3 ± 18.1
Widow	53.3 ± 19.2	49.3 ± 20.6	56.3 ± 24.2	37.4 ± 09.6	43.7 ± 26.0
Divorced	76.8 ± 12.6	73.0 ± 8.9	66.7 ± 23.6	73.4 ± 15.5	81.2 ± 8.9
Separated	72.6 ± 11.5	76.4 ± 15.0	68.7 ± 17.2	57.3 ± 11.0	79.2 ± 7.2
Never married	70.1 ± 15.4	68.3 ± 14.8	68.9 ± 15.7	55.5 ± 15.7	68.3 ± 17.2
*P value (F statistic)*	*< 0.001*	*< 0.001*	*< 0.001*	*< 0.001*	*< 0.001*
**Education**					
No education	58.3 ± 15.3	59.8 ± 16.9	66.8 ± 16.2	48.0 ± 15.7	58.0 ± 22.5
Informal education	63.2 ± 17.9	76.2 ± 13.5	74.4 ± 10.9	58.0 ± 14.0	70.7 ± 16.1
Can read / write	60.6 ± 14.9	64.0 ± 16.4	69.0 ± 16.1	52.5 ± 14.5	63.2 ± 18.3
Primary (up to grade 5)	65.5 ± 14.8	67.4 ± 14.6	72.7 ± 15.7	55.3 ± 14.2	67.7 ± 17.8
Secondary (up to grade 12)	68.0 ± 14.9	70.0 ± 14.7	72.3 ± 16.5	56.4 ± 15.2	69.4 ± 17.0
Tertiary (up to grade 16 or above)	67.4 ± 12.6	70.0 ± 13.7	73.9 ± 14.9	55.5 ± 15.4	74.7 ± 12.2
*P value (F statistic)*	*< 0.001*	*< 0.001*	*< 0.001*	*< 0.001*	*< 0.001*
**Disease**					
Physical disability	56.4 ± 21.4	62.2 ± 17.4	51.70 ± 13.5	55.4 ± 14.0	63.3 ± 22.9
Hypertension	61.3 ± 15.2	66.8 ± 15.9	57.0 ± 15.8	52.6 ± 17.4	66.7 ± 17.7
Diabetes	53.3 ± 15.7	62.5 ± 16.3	56.5 ± 15.6	47.8 ± 15.0	63.5 ± 16.8
Other	63.4 ± 15.3	65.6 ± 15.6	55.1 ± 14.2	54.1 ± 15.4	64.9 ± 19.8
None	68.2 ± 14.0	69.2 ± 14.4	58.20 ± 13.8	55.6 ± 14.3	70.0 ± 16.5
*P value (F statistic)*	*< 0.001*	*0.002*	*0.249*	*0.109*	*< 0.001*
**Employment status**					
Not working	63.4 ± 15.6	65.0 ± 17.1	70.4 ± 16.4	53.0 ± 15.8	64.5 ± 21.1
Working	66.0 ± 14.6	68.7 ± 13.7	72.3 ± 15.8	55.4 ± 14.7	69.0 ± 16.8
Retired	63.3 ± 18.9	62.7 ± 16.3	70.4 ± 15.8	54.3 ± 14.5	63.8 ± 21.1
*P value (F statistic)*	*0.034*	*< 0.001*	*0.195*	*0.047*	*< 0.001*
**Socioeconomic status**					
High	68.9 ± 13.7	72.3 ± 14.4	74.3 ± 15.6	60.4 ± 14.3	74.4 ± 15.5
Intermediate	65.3 ± 14.9	69.0 ± 13.1	74.1 ± 14.9	55.8 ± 13.6	68.7 ± 15.6
Low	61.5 ± 15.8	61.6 ± 16.0	67.4 ± 16.4	48.8 ± 15.0	60.4 ± 19.9
*P value (F statistic)*	*< 0.001*	*< 0.001*	*< 0.001*	*< 0.001*	*< 0.001*
**Social capital**					
High SC	66.2 ± 14.4	68.1 ± 13.4	73.3 ± 15.2	57.9 ± 13.6	68.9 ± 18.3
Moderate SC	65.2 ± 15.1	67.6 ± 15.2	71.8 ± 15.7	54.6 ± 14.7	67.7 ± 18.3
Low SC	60.7 ± 16.2	62.0 ± 17.6	67.1 ± 18.5	48.6 ± 17.7	60.8 ± 19.3
*P value (F statistic)*	*0.006*	*< 0.001*	*0.004*	*< 0.001*	*< 0.001*

*Using the 0–100 scoring method

### Multivariate linear regression model for nuclear family system

Table 5 presents the results of the multivariate linear regression model for nuclear family syste. In multivariate model, rural residence was negatively associated with physical − 2.55 units [95% CI: − 5.42 to − 0.33], psychological − 1.90 units [95% CI: − 4.9 to − 1.13] and environmental domains − 3.69 units [95% CI: − 7.27 to − 0.09]. Female gender was also negatively associated with QOL scores in physical, psychological and social relationship domains − 2.64 units [95% CI: − 4.59 to − 0.68], − 3.56 units [95% CI: − 5.5 to − 1.65] and − 1.92 units [95% CI: − 3.91 to − 0.07] respectively, no significant association was observed in environmental health domain. Increasing age was negatively associated with QOL scores. One-decade increase in age lead to 0.27 units [95% CI: − 0.35 to − 0.20], 0.15 units [95% CI: − 0.2 to − 0.06] and 0.12 units [95% CI: − 0.05 to − 0.12] reduction in scores of physical, psychological and relationship domains respectively. Presence of disease or disability led to significant decline in the QOL in physical domains. However, there was no significant association of disease and disability with QOL scores in other domains. QOL scores significantly declined with changing socio-economic status from high to low in all four domains. Social capital was also positively associated with QOL scores in all the domains.

**Table 5:** Multivariate linear regression analysis for physical, psychological, social and environmental health domains of nuclear family in Abbottabad, Pakistan (*n* = 1010)*

	Physical Domain	Psychological Domain	Relationship Domain	Environmental Domain
	β (95% CI)	β (95% CI)	β (95% CI)	β (95% CI)
*Fixed effects*				
**Residence**				
Urban	Ref.	Ref.	Ref.	Ref.
Rural	−2.55(−5.42 to 0.33)	−1.90(−4.9 to − 1.13)	–	−3.69(− 7.27 to −0.09)
**Gender**				
Male	Ref.	Ref.	Ref.	Ref.
Female	−2.64(−4.59 to − 0.68)	−3.56(− 5.5 to − 1.65)	− 1.92(− 3.91 to 0.07)	–
Age (Decades)	−0.27(− 0.35 to − 0.20)	−0.15(− 0.2 to) -0.06	−0.12(− 0.05 to 0.12)	–
**Disease**				
None	Ref.	Ref.	Ref.	Ref.
Physical disability	−8.78(− 16.1 to − 1.50)	–	–	–
Hypertension	− 3.58(−7.4 to 0.24)	–	–	–
Diabetes	− 13.0(− 19.0 to − 7.0)	–	–	–
Others	−4.75(− 7.02 to − 2.47)	–	–	–
**Socio-economic status**				
High	Ref.	Ref.	Ref.	Ref.
Intermediate	−3.86(−6.2 to − 1.54)	− 3.47(− 5.8 to − 1.15)	−0.58(− 3.10 to 1.95)	− 4.20(− 6.37 to − 2.02)
Low	−7.37(−9.73 to − 5.01)	−9.91(− 12.3 to − 7.5)	−6.54(− 9.89 to − 4.05)	− 9.89(− 12.1 to − 7.66)
**Social capital**	0.07(0.01 to 0.12)	0.12(0.07 to 0.18)	0.11(0.05 to 0.16)	0.18(0.13 to 0.23)
*Random-effects*				
**Level 1: Union Council**				
Gender	0.91(0.03 to 6.57)	0.99(0.30 to 3.99)	0.43(0.21 to 4.53)	2.36(1.31 to 4.22)
Age	0.02(0.00 to 6.64)	0.08(0.08 to 0.65)	0.06(0.02 to 0.18)	0.09(0.04 to 0.17)
Social capital	0.03(0.00 to 0.26)	0.08(0.06 to 0.17)	0.02(0.00 to 1.22)	0.01(0.00 to 1.39)
Constant	1.39(0.02 to 11.49)	0.04(0.00 to 0.90)	0.01(0.00 to 0.20)	0.00(0.00 to 0.03)
**Level 2: Cluster number**				
Constant	4.44(3.15 to 6.26)	4.45(4.07 to 5.86)	5.20(4.15 to 7.31)	5.26(4.04 to 6.84)
Residual	13.32(12.6 to 13.9)	10.14(11.8 to 12.9)	14.6(13.97 to 15.34)	12.1(11.51 to 12.8)

*Explanations: *Β* Beta coefficient, *CI* Confidence interval, *Ref* Reference group; The linear regression multivariate model adjusted for residence ownership, marital status, education and employment status in Physical domain and Psychological domain. Type of residence, residence ownership, marital status, education, employment status and disease for Social domain; Residence ownership, marital status, age, sex, education, employment status, and disease in Environmental domain; The short dashes (−) mean that the variable was removed by the stepwise deletion process in regression analysis

## Discussion

Our study is one of its kinds to assess the predictors of QOL domains in joint and nuclear families in Pakistan. We found that male gender, urban residence, younger age, higher socio-economic status and social capital were positive predictors in both types of family systems. Increasing age and presence of illness were associated with lower QOL scores in joint and nuclear families. Predictors were similar in for all domains of QOL across two types of families with few exceptions.

Family type has been reported to affect the mental and social wellbeing. A study from India reported that adolescents from joint family have better mental health compared to nuclear family [31]. Another study from India found no difference in the QOL scores between joint and nuclear family types except for social relationship domain where scores were significantly high for those living in nuclear families [18]. One study from Pakistan reported that elderly living in joint families had better social support and quality of life than those in nuclear families [32]. Another study from Japan reported that couples living as couples did not have any significant difference in the perceived physical and mental health while they were more likely to have severe hypertension compared to those in extended families [33].

Our study found higher scores for males in all four domains of QOL. This finding is similar to a study from India where females had lower scores [18]. A study from Kuwait also reported negative association of female gender with QOL scores [19]. A study from Iran also found that there were significant association between QOL and greatly varied by socio-demographic variables including gender [34]. These findings indicate that family members even within same family have different views about the family environment which could affect their QOL [35] and that of female members.

Ageing is associated with physical and mental changes in the body which affects the health and QOL. We found that increasing age was associated with decrease in the QOL scores in all domains except environmental domain in both types of families. Other studies have also reported similar association of age with QOL scores [18, 36]. With the increasing life expectancy countries will experience increasing proportion of elderly population. This calls for reorientations of systems and services to ensure healthy elderly.

Our study found significant association of socio-economic status with QOL scores in both types of families. Socio-economic status is associated with availability of resources and access to services which ultimately affect QOL. Studies on different populations have shown positive association of higher socio-economic status with higher scores in different domains of QOL [18, 34, 36]. Likewise, social capital was also associated with higher QOL scores in all domains a finding similar to studies from China and Malaysia [37, 38].

We found that presence of diseases was associated with lower scores in physical and psychological domains in joint families and with physical domain in nuclear families. Presence of any physical deformity or illness affect the physical and psychological health. Studies have consistently shown negative association of QOL with presence of diseases [19, 36, 39]. A study reported that people with mental and physical illness had significantly lower scores than healthy people in all three domains of QOL life except environmental domain [40].

We did not find any significant difference in the predictors of QOL among both family types. Our findings are interesting in a way that it is considered that QOL differs in both family systems and their predictors would also be different. There is a need to do further studies to explore this finding.

Our study is one of its kinds from Pakistan to assess the levels and predictors of QOL in joint and nuclear families from the randomly selected general population. We used robust statistical procedures and performed multi-level analysis to draw conclusions. However certain limitations need to be considered while interpreting the results of this study. First our sample was drawn from a single city which may limit the generalizability of our results. Second, questionnaire was administered by the interviewer which could introduce social desirability bias in the response. To minimize this, we ensured privacy during interviews and no other household member was allowed in the interview room. Thirdly, this was a cross-sectional study and temporal associations could not be ascertained with certainty and we cannot say surely whether the predictors of our study preceded the quality of life.

## Conclusion

Our study determined the levels and predictors of QOL scores of individuals in joint and nuclear families using validated WHO QOL BREF. Predictors were similar across both types of families. Male gender, urban residence, younger age, higher socio-economic status and social capital were positive predictors of QOL score while increasing age and presence of illness were associated with lower QOL scores among both family systems. These findings call for policy actions such as women empowerment, improvement in facilities in rural areas and poverty alleviation to improve quality of life. We also recommend further studies in different segments of population to further characterize the predictors of QOL.

## Data Availability

Corresponding author will provide all the relevant data used in this study upon request.

## Abbreviations

QOL: Quality of life
WHOQOL-BREF: World Health Organization Quality of Life Questionnaire Brief version
SD: Standard deviation
β: Beta coefficient
CI: Confidence Interval
Ref: Reference group
SC-IQ: Social Capital Integrated Questioners
